# Understanding Minds in Real-World Environments: Toward a Mobile Cognition Approach

**DOI:** 10.3389/fnhum.2016.00694

**Published:** 2017-01-12

**Authors:** Simon Ladouce, David I. Donaldson, Paul A. Dudchenko, Magdalena Ietswaart

**Affiliations:** Psychology, Faculty of Natural Sciences, University of StirlingStirling, UK

**Keywords:** mobile brain imaging, cognitive neuroscience, ecological validity, EEG, embodiment, situated cognition

## Abstract

There is a growing body of evidence that important aspects of human cognition have been marginalized, or overlooked, by traditional cognitive science. In particular, the use of laboratory-based experiments in which stimuli are artificial, and response options are fixed, inevitably results in findings that are less ecologically valid in relation to real-world behavior. In the present review we highlight the opportunities provided by a range of new mobile technologies that allow traditionally lab-bound measurements to now be collected during natural interactions with the world. We begin by outlining the theoretical support that mobile approaches receive from the development of embodied accounts of cognition, and we review the widening evidence that illustrates the importance of examining cognitive processes in their context. As we acknowledge, in practice, the development of mobile approaches brings with it fresh challenges, and will undoubtedly require innovation in paradigm design and analysis. If successful, however, the mobile cognition approach will offer novel insights in a range of areas, including understanding the cognitive processes underlying navigation through space and the role of attention during natural behavior. We argue that the development of real-world mobile cognition offers both increased ecological validity, and the opportunity to examine the interactions between perception, cognition and action—rather than examining each in isolation.

## A rationale for mobile real-world cognition

The human mind is a dynamic predictor that perceives, understands and acts within complex and ever-changing environments. To produce flexible and adaptive reactions that are relevant and appropriate to the individual's goals, the brain must integrate concurrent multi-modal sensory and motor signals, using continuous real-time feedback to guide the execution of on-going behavior. Despite this dynamic reality, however, the traditional approach to understanding human cognition has been the collection of empirical findings from experiments taking place in relatively static, often simulated, laboratory settings. Typically, participants sit or lie down, are given explicit and highly constrained instructions, and are required to attend to artificial stimuli whilst performing deliberately stereotyped responses. The strength of such an approach is the experimental control it affords; the cost, however, is a loss of real-world dimensionality, and perhaps, relevance. In the current article we present an alternative approach that capitalizes on recent technological developments to allow experimental work to be situated in the real world. This mobile cognition approach capitalizes on the ability to record brain activity (e.g., EEG) and body dynamics (e.g., eye movements) concurrent to natural behaviors. We believe that the emerging field of mobile cognition offers significant added value to traditional laboratory science, with particular implications for the translation of theoretical knowledge into impact. Before outlining this view, we first highlight the rationale for mobile cognition, which stems at least in part from dissatisfaction with assumptions underlying laboratory experiments: namely, that behavioral and neurobiological measures recorded under strictly controlled laboratory conditions accurately reflect the complexity of cognitive processing.

## The issue of ecological validity

Psychology has made substantial progress through the use of laboratory based experimentation; such has been the success of this largely reductionist approach that concerns have been expressed about it crowding out other fields within Cognitive Science (Gentner, 2010). However, despite its acknowledged success, right from the very beginning of the psychological investigation of cognition, concerns were raised about how ecologically valid many of the findings were. For example, as early as 1943, Brunswik expressed concern that cognitive psychology was heading toward the study of narrow and artificially isolated conditions that were not representative of the actual functioning of cognition (Brunswik, 1943). Similarly, significant debate around ecological validity was raised in the 1970s. Perhaps the most well-known advocate of this concern is Ulric Neisser, who argued (e.g., see Neisser, 1976) that assessing cognitive processes in an artificial environment would only enhance our understanding of those specific circumstances—but not necessarily generalize to real-world cognition.

More pointedly, Bronfenbrenner (1977) added that measuring restricted responses in artificial setups would generate behaviors that are in fact different from the behaviors displayed in a natural context. By this view, having participants sit at a computer looking at pixelated images or scenes may not allow researchers to fully characterize cognition—because the processes being engaged, or representations being accessed, occur in far more complex forms in real life. For example, researchers interested in recognition (as per witness identification scenarios in real life) typically present photographic images of people on computer screens—which are inevitably less rich than interacting with a real person. A striking example of the implications of this restriction can be found in the clinical case of agnosic patients (e.g., Goodale et al., 1991), who despite being unable to recognize or even describe the features (size, color, shape) of objects presented visually, are nonetheless able to appropriately adjust their movement toward these objects when interacting with them. This finding provided evidence that the internal representations associated with objects are accessed differently depending on the purpose of the output (vision for recognition vs. vision for action). A significant advantage of the mobile cognition approach is that it encourages researchers to investigate cognition in context, in relation to our natural interactions within the environment, rather than abstracted away from it.

Paradoxically, to our reading, one significant contribution to the problem of ecological validity has been the over enthusiastic pursuit of internal validity. Attempts to ensure that cognitive phenomena under investigation are being measured accurately and precisely (i.e., with high internal validity) have resulted in an ever-increasing drive toward greater resolution of data acquisition (e.g., greater number of electrodes during EEG recording, or finer-grained assessment of where a participant looks during eye-tracking). Furthermore, the concern for internal validity has contributed to the desire for ever improved signal to noise ratios during measurement and the removal of potential confounding variables via more and more artificial experimental set ups (Schmuckler, 2001). Equally, individual cognitive functions have gradually been studied at a greater and greater level of detail (i.e., specificity), with distinct processes being further and further subdivided in to sub processes of sub processes (e.g., declarative memory dividing into episodic and semantic memory, episodic memory dividing into recollection and familiarity, recollection into rate and precision, etc.). Whilst the push for greater internal validity is warranted, the increasing abstraction, isolation and focussing of measurements have, inevitably perhaps, contributed to an unintended reduction in ecological validity.

Of course, mobile cognition is not to replace laboratory work and the two must work in parallel and actively inform each other. While mobile techniques offer the unprecedented opportunity to investigate cognition in real-world context, they however do not currently compete with lab-based counterparts in terms of qualitative and quantitative features. The spatial resolution offered by a fMRI scanner will never be matched by a mobile fNIRS or EEG system. Furthermore, some research questions are better served by the experimental control afforded in laboratory research. Our view is that the mobile cognition approach will add considerable value to existing laboratory based cognitive neuroscience: indeed, there are many research questions that can only really be sensibly addressed in real-world contexts.

## Cognition is embodied

The lack of ecological validity in cognitive science has become even more of an issue with the emergence of evidence that cognition is embodied (for reviews, see Gallagher, 2005; Barsalou, 2008). Over the past 20 years it has become apparent that cognition is inherently reliant on its situated position in the environment. Chiel and Beer (1997) were one of the first to argue that understanding the interactions between brain, body and environment is crucial. The significance of this view has become more apparent through recent research that shows that bodily experience in fact shapes the way we process the environment. To be clear, the broad idea of interdependence between perception/action and the environment had already been emphasized by Gibson in his theory of affordances (1979), which states that the external information available to us is processed in relation to the opportunities for action that they provide. Although it was developed largely independently, embodiment theory also argues that cognition is for action, and furthermore that cognition is actually dependent on the bodily experience (e.g., Clark, 1999). Understanding cognition through abstract experimental paradigms that lack interaction with the environment, due to their artificial nature, therefore makes little sense from an embodied perspective.

Support for the embodiment of cognition can also be found in findings from modern day neuroscience. For example, evidence regarding neural plasticity collected over the last 20 years has made clear that brain and behavior are constantly shaped by our experience in interacting with the world. Even at the level of the functional organization of vision, it is clear that representations reflect top-down action potentialities rather than bottom-up sensory inputs (Bracci et al., 2012). One compelling example is provided by Thaler et al. (2011), who have shown that blind people can use echolocation to navigate in space (listening to the echo of clicks-sound to locate the reflection point), giving rise to functional restructuring of brain regions typically involved in vision. Perhaps the most famous example of how our behavior shapes our brain is the work on taxi drivers presenting with increased hippocampal size, which is thought to be due to their experience of navigating the maze of London streets (Maguire et al., 2000). Taken together, these finding highlight the fact that interaction with the surrounding world shapes brain structure and function. From this perspective, recent acceptance of cognition as being embodied emphasizes a problem inherent to traditional cognitive experiments—highly controlled and artificial experimental testing tends to separate cognition from natural bodily experiences. Furthermore, by separating cognition from the bodily experience we remove their intrinsic interaction that defines the human mind as an active agent.

**Figure 1:**
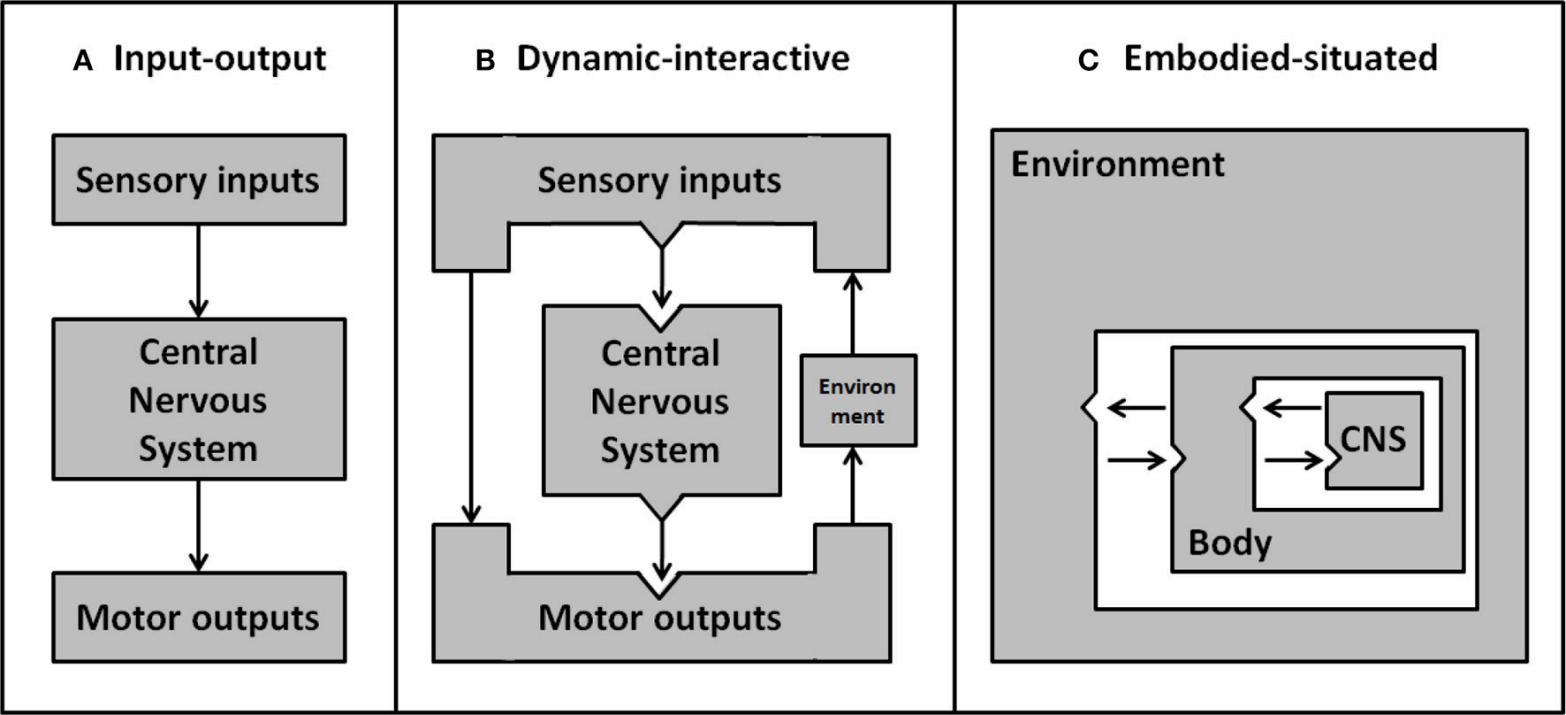
**Development of psychological models about the interactions between environment, body and central nervous system**. Early simple input-output models **(A)** ignore the environment and represent sensory inputs as being processed discretely to produce motor outputs. More sophisticated dynamic-interactive models **(B)** include environmental factors. By this account, direct feedback from motor output can interact with, and act on, the environment—resulting in a change to future sensory inputs. In embodied-situated models **(C)** the nervous system is embedded within the environment through the body. From this perspective input and output systems are integrated rather than discrete separable elements, and the nervous system is inherently linked with the environment—as parts of a dynamic system. Adapted from Chiel and Beer (1997).

The concept of situated cognition, stemming from the embodiment framework, postulates that our cognitive experience is dependent on the body's position in the environment. This interdependence implies that when embodied agents actualize their intentions they have to ensure their behavior accommodates the contingencies of the environment. In contrast, by looking at cognition through an ever-narrower lens, cognitive science has focused on the study of specific cognitive processes in isolation. Consequently, the complex interplay of perception, cognition and action has not generally been investigated (Beer, 2000). The isolated cognitive account of classic input-output models is clearly represented by the idea of minds as machines, decoding sensory inputs (perception) to then deliver output commands (behavior) to the body (see Figure 1A). Whilst the unilateral direction from input to output implies a major bottom-up influence (and questions the very existence of self-motivated behavior), there is now abundant evidence of top-down and dynamic influences on the selection and processing of features relevant to the ongoing task (Henderson, 2007). For example, the execution of naturalistic goal-oriented tasks induces anticipatory eye movements toward relevant affordances (e.g., Pelz and Canosa, 2001; Mennie et al., 2007; Hayhoe et al., 2012). Another example of this dynamic interplay between input and output is the integration of visuomotor feedback, allowing for online correction of movements to sudden changes of the environment (e.g., adaptive reaching movements to avoid obstacles; Chapman and Goodale, 2010). This body of evidence emphasizes that perception and action are interdependent and that bodily experience influences the way we process (input) and act on (output) the environment (see Figure 1B). By assessing only one aspect in isolation, as previously done in cognitive research, the dynamic interplay between cognitive functions cannot be captured. Ultimately, therefore, it will be necessary to integrate concurrent measurement of input and output if we wish to account for the dynamic interplay of cognitive functions in the face of a rich and dynamic real-world environment (see Figure 1C).

## Brain states differ during movement

A growing animal literature demonstrates the fundamental importance of interactions between brain, body and environment. For example, in the mouse hippocampus, 75% of place cells (neurons which encode specific locations in the animal's environment) show a significant decrease in firing when the mouse is prevented from moving (Chen et al., 2013). Further, in rats, changes in theta power associated with ambulation (McFarland et al., 1975; Long et al., 2014) are also modulated by the anticipation and initiation of goal-directed instrumental behavior (Wyble et al., 2004; Sinnamon, 2006). In monkeys, the hippocampus is essential for a task where the animal must walk to a to-be-remembered location (Hampton et al., 2004), but it is not needed when the same type of memory task is performed while the animal is seated (Malkova and Mishkin, 2003). Importantly, the inter-dependence between locomotion and cognition is bi-directional: brain dynamics in Drosophila suggest that the processing of visual information is different in flight compared to resting state (Maimon et al., 2010). To be clear, existing data reveals an inter-dependence between cognition and the exploration of the environment that emphasizes the importance of understanding cognition in real-world contexts (suggesting that even studies in virtual reality will be insufficient to characterize cognitive processes as they support everyday behavior). Strikingly, emerging findings support the claims made by Bronfenbrenner (1977) that cognitive responses measured in artificial experimental conditions are different from those in natural exploration, and furthermore that ecological validity is not just desirable but essential if we are to fully understanding cognition.

Whilst existing evidence clearly demonstrates that the neural correlates of visual perception or locomotion can be altered by context, one reading of these data is that they only argue for sensitivity in sensory or motor systems, rather than in association cortex linked to higher order cognitive processing. However, the dual-task literature demonstrates that introduction of gait and balance control has a significant interfering effect on higher-order cognitive processes such as executive functions (i.e., inhibition, divided attention), verbal fluency, decision-making and working memory (for a review, see Al-Yahya et al., 2011). As well as linking bodily changes to cognitive performance, these data also question the validity of static single-task experiments in particular, because everyday life necessarily involves considerable cognitive-motor multitasking. In short, embodied cognition theory argues that sensory, motor and cognitive processing are interdependent, and evidence supports this claim, providing a strong rationale for studying cognition in motion or while engaged in natural motor tasks (Schaefer, 2014).

## The emergence of mobile methods

**Figure 2:**
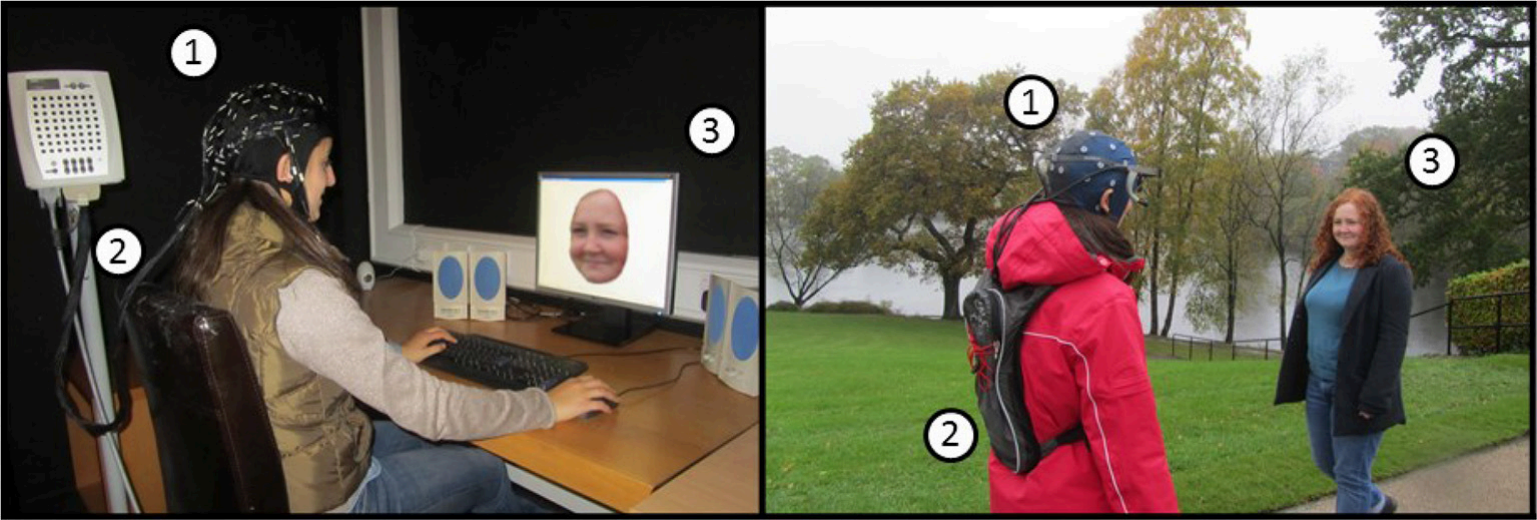
**An example of the differences between a laboratory-based (left) and a mobile real-world (right) experimental setup using EEG**. (1) EEG sensors, (2) Amplifier and data storage unit, (3) Stimulus presentation. Using the example of a classic face recognition paradigm, this figure illustrates the typical laboratory setup (left) in contrast to recording of real-world face recognition (right). In the latter, faces are presented in-context, while the participants are behaving naturally experiencing a real-world environment. Note that event triggers are also implemented differently, i.e., based on computer controlled timing of stimulus presentation or on behavioral response in the laboratory, in contrast to event registration based on natural behavior in relation to stimuli in the scene (e.g., as assessed by fixation points recorded with a head mounted eye tracking device). Mobile brain imaging of neural activity with behavioral measurements permits the study of cognition underlying everyday life.

Despite long standing awareness of the issue of ecological validity, and the recent accumulation of evidence highlighting the need for a real-world approach in cognitive research (e.g., Clark, 1997; Smilek et al., 2006; Williams and Long, 2015), the issue has not been widely addressed in practice. Indeed, on the whole, these concerns are only incidentally acknowledged in the literature (Sbordone, 1996; Burgess et al., 1998; Chaytor and Schmitter-Edgecombe, 2003; Spooner and Pachana, 2006; Williams and Long, 2015). At least in part, this inertia can be explained by a lack of satisfactory solutions to conduct ecologically valid studies while maintaining scientific rigor and high levels of data quality. Studying cognition in the real-world demands a combination of technical and methodological requirements (Makeig et al., 2009; Reis et al., 2014), including portable devices that can operate with minimal noise, whilst also developing paradigms that retain an adequate degree of experimental control (see Figure 2).

Recent technological developments have led to the advancement of portability of traditional brain imaging and behavioral measurement techniques. Research techniques that were previously restricted to laboratory settings due to hardware limitations (e.g., weight, size, battery life) have become fully portable. Whilst it is beyond the scope of the current article to exhaustively review available equipment (for a comprehensive review see Gramann et al., 2011), here we highlight a number of key developments that illustrate the changing landscape of tools with which cognition can be measured.

The last decade has seen the emergence of compact, lightweight, non-invasive and wireless brain imaging hardware that do not hinder everyday movements, and yet still provides accurate recordings of brain cortical dynamics. Indeed, mobile iterations of electroencephalography (EEG) and near-infrared spectroscopy (fNIRS) systems have evolved rapidly to closely match the standards of high-density laboratory versions in both spatial and temporal resolution (Gramann et al., 2014b). More than just recording of brain activity in motion, mobile techniques allow brain processes to be captured “on the go,” in relation to natural behaviors in real-world environments (such as navigating the streets of London either on foot or as a taxi driver, choosing products in a shop, putting a golf ball, or an elderly person getting up and moving about). Importantly, early proof-of-concept studies have reported successful recording of classic EEG components during motion (Gramann et al., 2010; Debener et al., 2012; Severens et al., 2012) and the detection of task-related changes in hemoglobin concentration using wearable fNIRS (Koenraadt et al., 2014; Piper et al., 2014).

While a range of mobile imaging techniques exists, it is important to recognize that they each offer particular strengths. Multi-channel fNIRS offers a good spatial resolution over a delimited cortical surface, describing neuro-vascular changes at a cortical level, but this technique lacks the temporal resolution required to investigate fast cognitive processes. In contrast, the high temporal resolution of EEG can reveal rapid changes in electro-cortical activity related to the ever-changing demands of real-world cognition. An additional advantage of mobile EEG as a neuroimaging tool is that EEG has been used extensively in laboratory-based settings, providing a background of information against which mobile cognition data can be benchmarked. Nevertheless, as discussed below under “current challenges,” there are considerable methodological issues associated with the application of mobile brain imaging—and an important element of current research is to overcome these remaining issues and demonstrate the viability of mobile neuroimaging.

**Figure 3:**
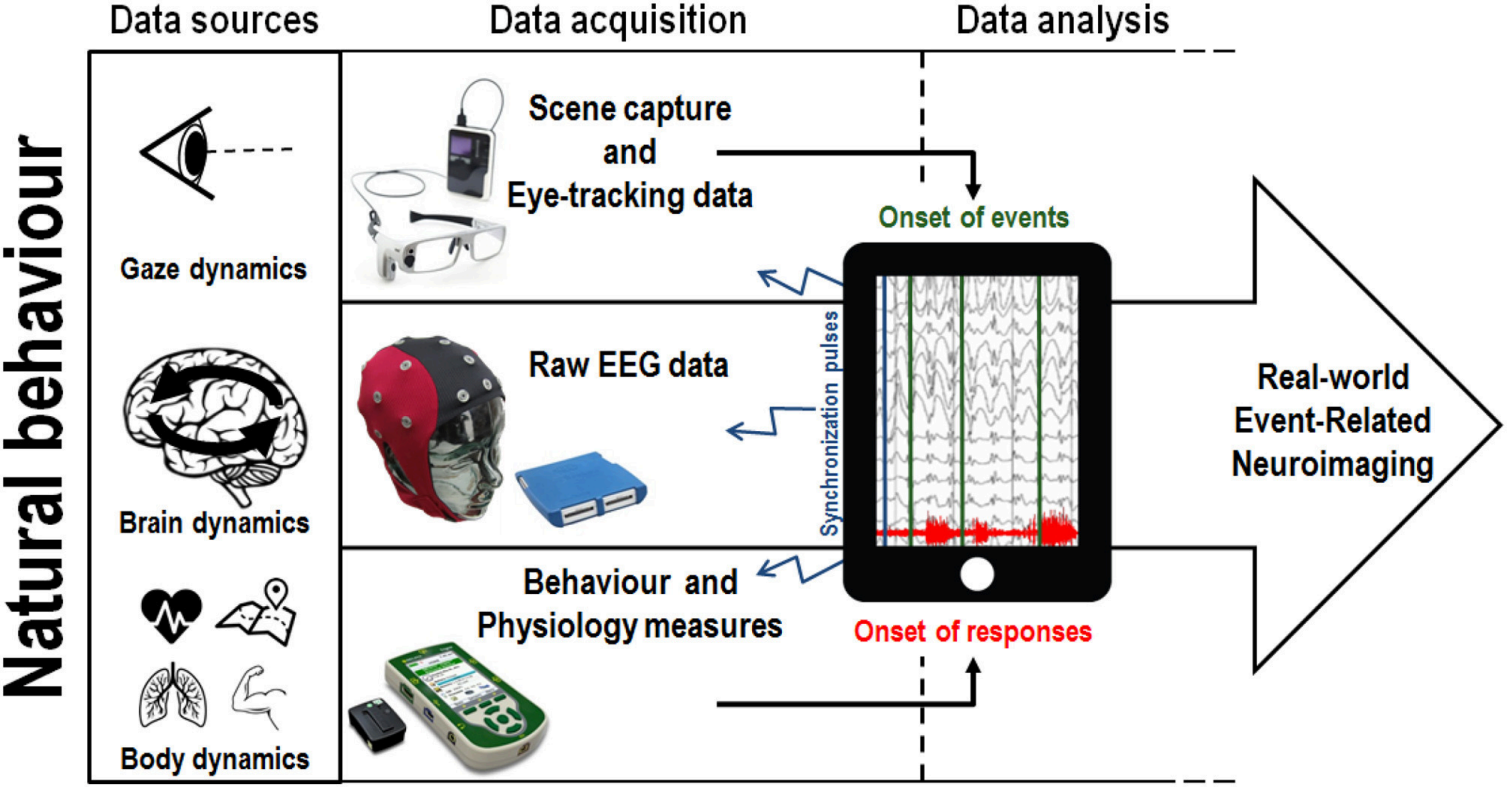
**An illustration of one potential implementation of the mobile cognition approach to real-world brain imaging**. Natural behavior provides multiple sources of data, recorded concurrently, allowing the integration of mobile eye-tracking and body dynamics measurement with mobile electroencephalography (EEG). A concrete example of the application of this integrated approach can be found in a shopping situation where fixations on target objects will be use to timestamp the EEG and proceed to the classification of brain responses. Conversely, in a sport scenario, the onset of a specific goal-oriented sporting behavior will be used to extract meaningful information from the continuous EEG trace. We note one significant technical challenge associated with this multi-methods approach: in practice the simultaneous synchronization of data acquisition across devices is non-trivial because each individual measure has typically been developed and used in isolation.

Methodologies used to capture behavioral responses have evolved from simple movement measures (button presses, singular body part acceleration) to the measurement of natural whole body kinematics (Aminian and Najafi, 2004). In particular, motion capture systems have become increasingly portable, allowing the positioning of multiple independent wireless sensors on a single participant, such that movement can be recorded unconstrained (Lim et al., 2011; Marin-Perianu et al., 2013). While most current motion capture solutions still require external cameras to track the position of sensors (thereby restricting the recording area), camera-less motion-capture alternatives using networks of inertial and magnetic sensors methods (exoskeleton suits composed of accelerometers, gyroscopes and magnetic sensors) may be used to detect changes in position, orientation and acceleration of body parts, allowing kinematics to be tracked in complete autonomy of external stationary devices (Zhu and Zhou, 2004; Luinge and Veltink, 2005; Roetenberg et al., 2007). Similar to mobile EEG, wireless electromyography (EMG) systems have also been developed to record muscular activity in complete freedom of movement (Roy et al., 2013). From a mobile cognition perspective, the integration of high resolution behavioral measurements with real-world mobile brain imaging make it possible to study the cognitive markers related to natural behavior in relevant environments. Indeed, in this context, one important role of body kinematics and EMG data may be to define the onset of behavioral responses and motor outputs (see Figure 3).

Recent studies have applied this multi-modal brain and body imaging approach to characterize brain dynamics related to upright walking through the integration of simultaneously recorded multi-modal data streams (Bulea et al., 2013). By using gait dynamics such as heel strikes to time-lock continuous EEG recordings, Gwin et al. (2011) have reported increased power spectral activity in the left and right sensorimotor cortex during contralateral foot suspension in subjects walking at a steady pace on a treadmill, suggesting increased cortical involvement related to visuo-motor integration and error monitoring. More recently, Wagner et al. (2016) reported different patterns of power spectral activity reflecting movement initiation and execution (Mu and Beta desynchronization in sensorimotor and parietal cortex) and motor control and inhibition (increased frontal Beta power) during a gait adaptation task. These important early findings demonstrate the feasibility of characterizing modulations of EEG activity in relation to body dynamics through the integration of brain and body measurements. Nonetheless, whilst treadmill studies are undeniably important for establishing the feasibility of recording brain activity in motion, they inherently remain lab-based demonstrations rather than real-world applications.

Mobile technological development has also occurred for eye-trackers, which have developed into wearable devices that can track gaze dynamics during head rotations and full body motion (Pelz et al., 2000; Babcock and Pelz, 2004). As a result, mobile eye-trackers are now able to provide insight on the deployment of attention in real environments. Additionally, eye-tracking glasses are now typically equipped with a high-resolution camera enabling synchronous audio-visual recording. An increasing number of studies are using head-mounted eye-tracking devices to record natural visual exploration during natural behavior (e.g., see Hayhoe and Ballard, 2005). Mobile eye tracking has been applied to the investigation of visual memory and motor planning of everyday-life behaviors (e.g., Pelz and Canosa, 2001; Mennie et al., 2007), predictive eye movements in sports (e.g., in squash, Hayhoe et al., 2012), in developmental research (e.g., see Franchak et al., 2010), and has been adopted in the context of marketing research (e.g., see Gidlöf et al., 2013). Here we highlight one important consequence of the development of mobile EEG and eye-tracking: in future the combination of gaze dynamics and first-person scene capture will enable the timestamping of visual events either based on fixations (Baccino and Manunta, 2005) or saccades (Jagla et al., 2007) in event-related real-world brain imaging (see Figure 3). Taken together, these techniques are beginning to allow us to extract brain and gaze dynamics related to real-time, every-day, real-world cognitive processing.

## Current challenges

For the first time these mobile devices are enabling researchers to record behavioral, neural and physiological markers that reflect cognitive processing as it occurs in natural contexts, while subjects are freely exploring and interacting with their environment (Makeig et al., 2009). These new technologies allow researchers to investigate cognition in an ecologically valid and integrated manner that is more representative of the intrinsic interdependence of perception, cognition and action. But although mobile equipment is typically smaller and lighter in weight (and often wireless) than the static equivalent, they nonetheless remain subject to similar constraints in terms of data acquisition and analysis and associated methodological challenges. Furthermore, there are a number of additional methodological challenges associated with mobile cognition that require innovation.

One critical concern is the optimization of the signal-to-noise ratio, and the fact that mobile participation inevitably produces more noise (Gwin et al., 2010). Considering the complexity of disentangling neural signal from noise, traditionally researchers have opted to act pre-emptively by minimizing the potential for “artifacts” (data unrelated to the cognitive process under investigation). For example, during EEG data acquisition, eye movements, along with facial and neck muscle activity, are prevalent sources of noise. Attempts to minimize the impact of artifacts has typically translated into avoidance techniques, instructing participants to remain still and suppress any movement not directly related to the performance of the experimental task, and requiring participants to inhibit natural reflexes such as blinks and swallowing (Picton et al., 2000). Within the mobile cognition approach, however, the aspiration is to allow natural unconstrained behavior—precluding the use of avoidance techniques.

The introduction of motion in itself requires the wholesale re-evaluation of established practices in laboratory-based cognitive research. Success may therefore require innovation in experimental design, data processing methods, and analysis techniques to reveal the patterns hidden within real world brain dynamics. Advanced processing methods to deal with the inevitable motion-related artifacts are in development. Independent Components Analysis (ICA) (Makeig et al., 1996), which involves the statistical linear decomposition of EEG data into maximally independent components, can be applied to the identification and dissociation of non-brain signals (e.g., line noise, mechanical artifacts) from cognitive brain activity, eye movements and muscular activity (Delorme et al., 2007).

As noted above, mobile EEG studies have successfully addressed motion-related artifacts present in data recorded during high physical activities such as running on a treadmill (Gwin et al., 2010). Equally impressively, recent study successfully demonstrated the feasibility of parsing non-brain from brain signals in subjects cycling in a natural environment (Zink et al., 2016). Moreover, advanced EEG data analyses can be applied toward signal source localization based on the reconstruction of equivalent dipoles of independent components (Gramann et al., 2010; Wagner et al., 2016). However, EEG source modeling methods are essentially based on computational derivations and therefore require a feed of high-dimensional EEG data (i.e., 120+ channels) in order to reach sufficient approximations of signals' origins. While offering an interesting option for brain signal source estimation, high density EEG is still impractical for the use in real-world environments. The high-density set-up required for this approach are not truly mobile at present: for example the studies by Gramann et al. (2010) and Gwin et al. (2010) took place on a treadmill with EEG cables suspended from the ceiling. Therefore, although such solutions allow recording of brain activity in motion, they are not yet useable in truly real-world settings.

Even if high-density EEG recording was developed to allow free movement through natural spaces, advanced signal processing methods would still require sufficient amount of data points. The same is true for the most popular approach of isolating brain signals and their dynamics from the ambient noise in the raw data: time-locked averaging of many trials linked to hypothetical cognitive processes. This approach yields Event-Related Potentials (ERP), time-locked deflections in the EEG trace reflecting sensory, cognitive and motor processes in the time domain at a milliseconds scale (Luck, 2005). Brain signals of interest (those related to events such stimulus presentation and behavioral responses) are generally uncovered by canceling out unrelated signals through summation and averaging of multiple trials. A real concern when moving into mobile data settings is that it may be impractical (and in some cases unnatural) to record the required numbers of repetitions of a specific event. On the other hand, a distinct advantage of real-world mobile methods is the ability to record data over longer periods (e.g., during home-based monitoring of patients), making collection and characterization of larger scale single participant data sets a real innovation.

The example of high-density recording as a solution to current challenges also flags up the issue of fully equipped participants' appearance potentially defeating the purpose of increasing ecological validity—by rendering subjects more self-conscious about the experiment and affecting their real-world interactions. Mobile EEG systems using dry sensors may be a convenient and user-friendly solution to reduce preparation time, which could make mobile EEG more accessible to patient and consumer based applications (Zander et al., 2011; Chi et al., 2012; Dias et al., 2012). However, state of the art dry electrodes EEG systems still do not match electrodes with conductive gel applied in terms of data acquisition quality, and are less comfortable for the subjects (Oliveira et al., 2016). From an aesthetic point of view, recent studies have proposed in ear EEG sensors (Looney et al., 2012; Kidmose et al., 2013; Mikkelsen et al., 2015; Goverdovsky et al., 2016), around-the-ear electrodes grids (Debener et al., 2015; Bleichner et al., 2016; Mirkovic et al., 2016), or a baseball cap fitted with electrodes (Bleichner et al., 2015). Future developments in mobile brain imaging should therefore aim to increase the ease of use, discretion and comfort of the sensors while maximizing data quality in order to be successfully applicable in real-world settings.

Given their high temporal resolution, ERPs have been invaluable in the investigation of the time-course of cognitive processes involved in the integration of sensory inputs and motor output in the face of a dynamic reality. The major practical issue with mobile ERPs lies in the acquisition of accurate timings of such events, time-stamping the EEG trace based on stimulus presentation and behavioral responses. Whilst the time-locking of events of interest has been facilitated through the use of computerized paradigms in laboratories settings, acquisition of the precise timing of events of interest is much more complex in a natural environment. This issue is of critical importance since event-related components are investigated at a millisecond scale. A recent study by Jungnickel and Gramann (2016) demonstrates the feasibility of recording brain activity time-locked to physical interaction with dynamically moving objects. In this case the definition of movement onset was based on velocity features of behavioral responses, recorded through motion capture. The results revealed faster behavioral response times and increased neural response (P300 following target stimuli) during physical pointing, in comparison to a classic button press condition. Jungnickel and Gramann interpret these results as suggesting dynamic integration of perceptual inputs, along with the execution of complex motor outputs, lead to higher computational efforts related to embodied cognitive processes.

Another classic approach in EEG research is the investigation of changes in the frequency domain. Through spectral density estimation methods, it is possible to characterize frequency bands contribution to recorded data. Power Spectral Density (PSD) estimation methods (e.g., variants of Fourier Transform) characterize frequency bands contributions to whole epochs signals. However, this stationary approach poorly represents dynamic changes over time, central to mobile EEG. More recently however, time/frequency analyses have given insights into Event-Related Spectral Perturbations (ERSP) allowing the characterization of power spectral modulations in the time domain (Makeig et al., 2004). In addition, the characterization of interactions between remote cell assemblies could provide insight as to how different parts of the brain work together to bind multimodal information to create a coherent perceptual experience. Traveling waves analyses assess the propagation of brain signals in terms of mode and velocity, to uncover local networks connections from global fields activity (Nunez and Srinivasan, 2006). For example, recent evidence from human electrocortigraphy (ECoG) supports the idea that theta oscillations related to working memory are traveling waves, showing a spatial propagation across the hippocampus (Zhang and Jacobs, 2015). Based on a theoretical connectionist model of cognitive functions, traveling waves applied to data acquired during natural behaviors may yield insight about brain-wide cognitive networks underlying everyday life cognition.

Given the range of analytic techniques that could be applied to real-world data an important development will be the synchronization of concurrent behavioral measurements to brain dynamics in mobile settings, such as simultaneous recording and integration of mobile EEG and eye-tracking data. Merging of eye-tracking data (along with first-person audio-visual recording) would provide information about the actual timing of engagement with real-world stimuli. For example, initial fixations on an object or person can in principle be used to generate *post-hoc* timestamps for the analysis of EEG data. To our knowledge, there is currently no scientific publication reporting such integration of mobile eye-tracking and EEG data in a real-world environment. Even though eye-movements related potentials have been used in laboratories settings, specific technical challenges inherent to mobile eye-tracking and mobile EEG might still impede the integration of both techniques. Eye-tracking data acquired in laboratories setups is usually based on a fixed reference frame (e.g., computer screen) which allows for the segmentation of this two-dimensional frame in pre-defined Regions of Interests (ROI). This segmentation facilitates the quantification of gaze dynamics across meaningful parts of the visual display. In the case of mobile eye-tracking recording, this frame of reference is dynamically affected by the subject's displacement across the three-dimensional planes of the environment. Therefore, the definition of ROIs in mobile environment is a significant challenge to the analysis of mobile eye-tracking data. Current options to address this issue reside in the use of optical pattern barcodes (i.e., QR codes) or infrared-based markers placed in the environment to delimit ROI. However, this approach requires ROI to be defined *a priori* and any gaze dynamics recorded outside these areas still have to be annotated and processed manually. A potential solution for the processing and analysis of mobile eye-movement data is the use of automatic mapping of the video stream through object recognition algorithms (Brône et al., 2011). Even with these solutions to ROI definition, recording eye movements in a three-dimensional environment also requires accounting for depth, which has not been resolved yet by current mobile eye-tracking systems. Since the calibration procedure is performed on arrays of elements presented at a predefined distance, eye fixations on elements beyond the range of the calibration are usually poorly tracked by current systems and represent another major issue in the study of visual exploration in natural contexts.

As the preceding discussion highlights, there are real practical challenges in developing mobile approaches. We anticipate that the mobile cognition approach will make such multi-methods data collection more attractive across a range of measures, allowing the study of natural behaviors (Gramann et al., 2014a) in context and thus providing greater ecological validity in the process (see Figure 3). The development of mobile brain imaging methods follows the same dichotomy as the current state of mobile brain imaging literature. While the initial push toward mobile neuroimaging sensors was largely driven by brain-computer interfaces applications aiming to maximize the online classification of signal components at minimal cost in terms of data acquisition requirements, a considerable number of mobile brain imaging studies have turned toward mobile systems with more sensors, in order to record the high-dimensional data required to perform advanced signal analyses. Consequently, there is now a large spectrum of hardware and software solutions, which vary in terms of their ease of setup, quality of data acquisition and cost. While the on-going competition between manufacturers may be regarded as a healthy drive in the improvement of mobile brain imaging technologies, it also seems likely that discrepancies in terms of conceptual and methodological standards (related to both data acquisition and data analysis) may slow down the progression of the field toward standard practices.

One important attempt at developing standardized frameworks for mobile technology comes from open-source initiatives designed to support the processing and analysis of mobile brain and body imaging data and facilitate their integration (e.g., MoBILAB; Ojeda et al., 2014 and Lab Streaming Layer; Kothe, 2014). While these frameworks allow for the recording and processing of multimodal data, the exact synchronization of data streams remains problematic due to current hardware limitations. For example, differences in terms of refresh rate across mobile techniques can lead to inconsistencies (or jittering) in the time-stamping of EEG data. Notably, even though current mobile eye-tracking devices now offer up to 120 Hz sampling rates, this is still insufficient to define the onset of visual events in the EEG trace with enough precision to carry milliseconds scale analyses in the time domain. Thus, whilst existing data processing schemes offer clear benefits for a mobile cognition approach, at present, the acquisition of events markers remains a non-trivial challenge to the investigation of cognition in the real-world.

Besides the increased noise that inevitably accompanies cognition in motion, controlling for confounding variables will also be a significant challenge in everyday environments. Due to the rich and unpredictable nature of the outside world, inconsistencies may arise across conditions and between subjects. Whilst resolution of most of the aforementioned issues will build upon future technical improvements of the techniques, inventive experimental designs and methodological compromises will also be of critical importance to translate cognitive research into the real-world.

## Current state and future directions

Given the novelty of mobile technologies it is perhaps unsurprising to discover that the current state of the literature mainly consisting in proof-of-concept experiments assessing the feasibility of brain imaging in motion. Most published studies have worked toward the validation of mobile techniques through the replication of paradigms known to reliably elicit specific neural signals, such as well-known ERP components. These technically oriented studies have provided evidence that portable brain imaging can reach comparable levels of accuracy as traditional stationary devices within the same stationary laboratory setup (Gargiulo et al., 2008; Dias et al., 2012; Liao et al., 2012; De Vos et al., 2014b, and also during treadmill walking (Gramann et al., 2010; Severens et al., 2012; Lin et al., 2014). This body of research has pinpointed technical (e.g., ensuring the necessary sensor connectivity during whole-body movements), methodological (e.g., time-stamping of events in real-life situations) and mathematical questions (e.g., tackling motion artifacts) posed by mobile brain imaging (for a review, see Reis et al., 2014). Hardware and software solutions have been developed in response to these issues of concern (e.g., MoBI; Gramann et al., 2014b), providing a solid framework to progress to the next step: addressing actual cognitive questions in natural environments.

Interest in mobile cognition can be found across a number of different fields in neuroscience—the relevance of real-world cognition has been highlighted for sport (Park et al., 2015; Cheron et al., 2016), ergonomics (Mehta and Parasuraman, 2013), dual-task paradigms (De Sanctis et al., 2014), spatial cognition (Mavros et al., 2016) and mental imagery (Kranczioch et al., 2014). Increasing numbers of studies are investigating cognitive processes during full-body motion in the real-world. Perhaps the clearest example to date is provided by Debener et al. (2012), who used an auditory oddball task to elicit P300 ERP effects. The P300 is a well-characterized and much studied neural marker of attention, found during the presentations of a series of frequent distractors vs. infrequent odd-ball targets (for a review see Polich, 2007). Debener et al. (2012) recorded the P300 in a seated-indoor condition vs. an outdoor-walking condition. An attenuation of the P300 ERP amplitude was reported in the walking condition in comparison to the sitting condition. While classification rates of single-trial ERPs were above chance levels for both conditions, the signal-to-noise ratio (SNR) was lower in the walking condition, suggesting an increased amount of noise in that condition. Whether these differences were a consequence of residual noise or due to a reallocation of cognitive processing resources in the outdoor-walking condition remained open for future investigations. A follow-up study compared outdoor-walking with being seated outdoors, finding equivalent P300 effects in each case (De Vos et al., 2014a). Importantly, a similar degree of noise was found in walking and seated conditions, suggesting that the muscular activity involved in walking did not result in increased movement-related noise. Furthermore, Zink et al. (2016) have reported a decrease in P300 amplitude during an outdoor cycling condition in comparison to a fixed bike conditions. The increased cognitive load related to natural real-life behaviors appeared to be a major factor contributing to the difference observed in ERP waveforms between conditions.

Why these natural behaviors (i.e., walking, cycling) should reduce attention (as indexed by changes in the magnitude of the P300) compared to being seated indoors remains unexplained—and an important question for future research. Regardless, and more relevant here, the studies by Debener and colleagues demonstrate the feasibility of truly mobile EEG recording in the real-world.

**Figure 4:**
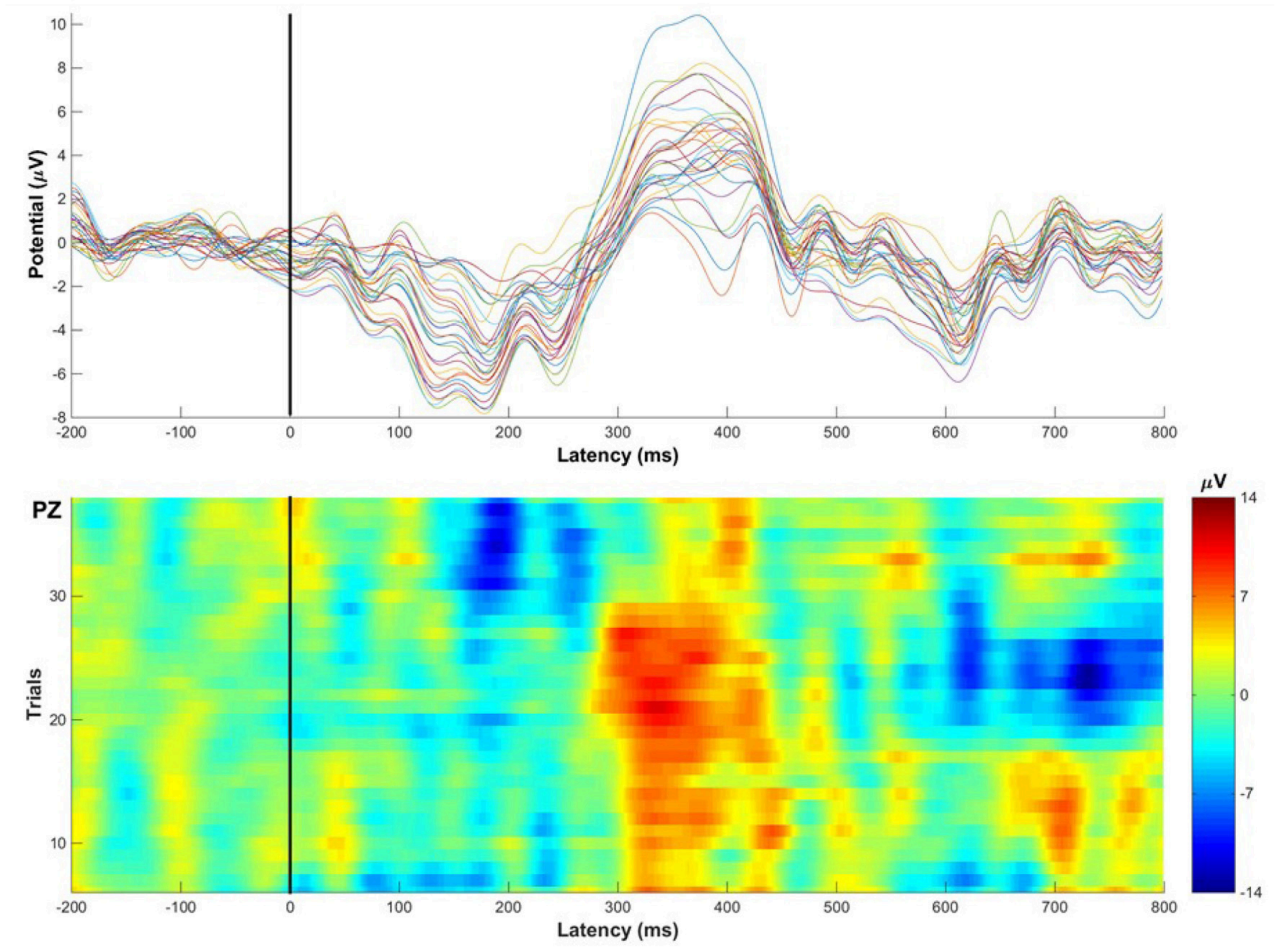
**Illustrative single-subject ERP example recorded while the participant walked around the corridors of Stirling University performing an auditory oddball task (eliciting the classic P300 Event-Related Potential)**. Top: Average ERP waveforms across 32 channels, the P300 amplitude is most prominent at mid-parietal electrode sites, showing the classic P300 scalp distribution. Bottom: 36 single-trial Event-Related Potentials of target stimuli classically recorded at Pz electrode displaying consistent amplitude peaks 300 ms after stimulus onset. Examples of mobile EEG findings can be found in the literature (e.g., Debener et al., 2012; Zink et al., 2016). This figure of single-subject raw data provides a visual demonstration to show that ERPs can be reliably recorded across trials and electrodes during locomotion in the real-world.

**Figure 5:**
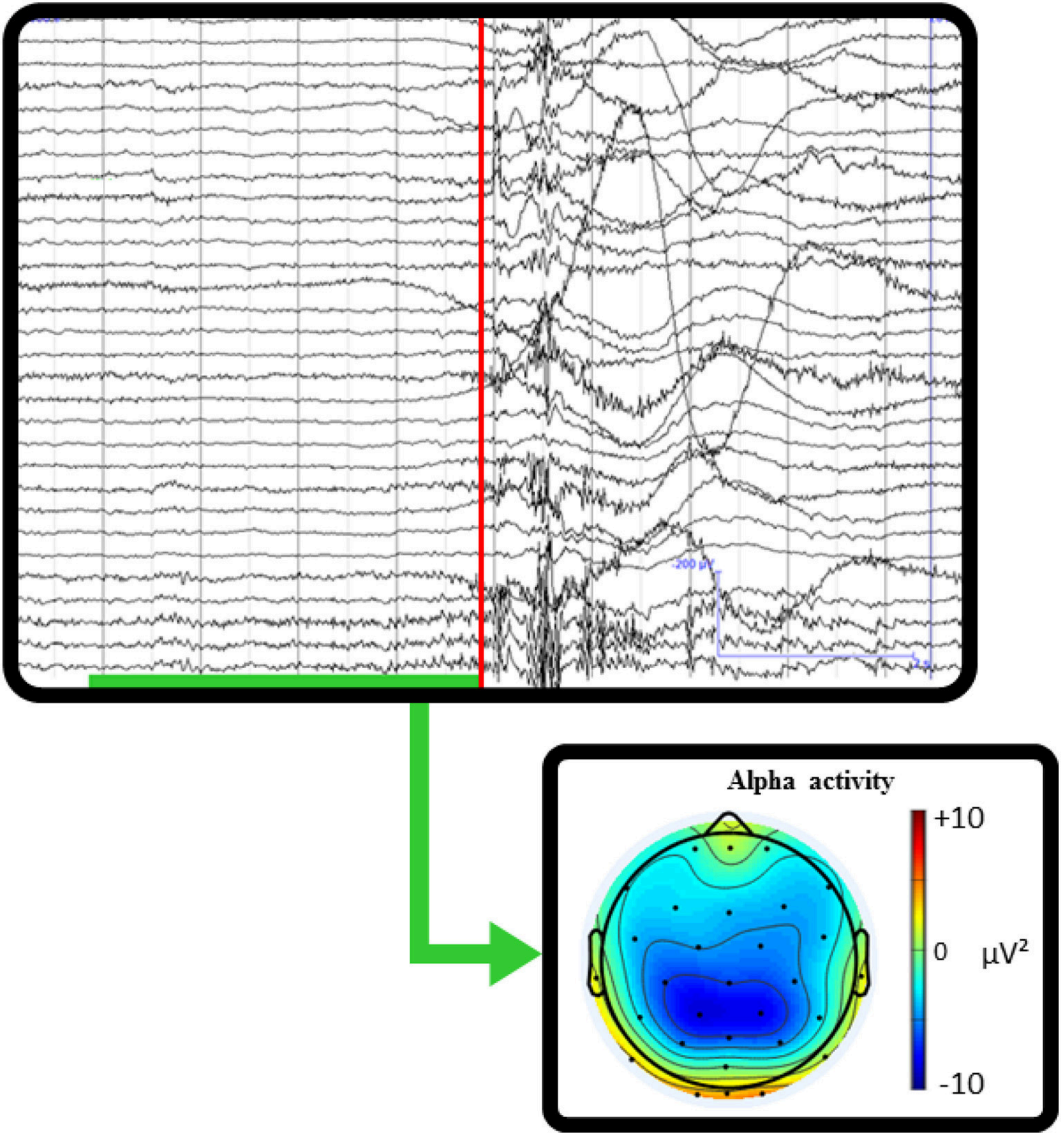
**Single-subject real-world example of mobile EEG recording while taking a penalty kick**. The red line marks the timing at which the ball was hit. The green underlined time interval represents where the participant is mobile, stepping up to kick the ball. The topographic scalp map shows the averaged alpha activity during the pre-shot interval before kicking the ball. This example recording illustrates how the integration of behavioral markers with mobile brain imaging could allow insight into cognitive processing related to natural behaviors. The quantification of changes in power spectral activity related to the execution of goal-oriented actions would provide information about the cognitive aspects related to real-world sporting behaviors.

Whilst there is relatively little published data thus far, existing studies have aimed primarily at testing and validating mobile cognition methods using traditional well-established paradigms from the lab setting as benchmarks (Oliveira et al., 2016). Critically, the evidence to date provides strong proof of concept regarding the basic feasibility of a mobile cognition approach (e.g., Figure 4). It is important to recognize however, that whilst the demonstration of proof of concept was necessary and provides confidence in the mobile methods going forwards, it did not directly contribute to further our understanding of real-life cognition, *per se*. Moreover, Kingstone et al. (2008) argue that the legacy of laboratory-based practices and experimental protocols may induce bias in the capture of the expression of human cognition in complex environments. For further progress to be made, future mobile cognition research must not focus solely on mimicking lab-based research, but should also investigate human cognition from an embodied, integrated and ecological perspective—assessing more naturalistic real-world behavior (e.g., Figure 5).

## Implications

Our view is that the mobile cognition approach will add considerable value to existing laboratory based cognitive neuroscience: indeed, there are many research questions that can only really be sensibly addressed in real-world contexts. Take, for example, the study of spatial navigation. To date, in humans, the examination of neural correlates of spatial cognition has been limited to a small subset of the questions that are examined in non-human animals. Many studies in rodents allow free-movement through space, whereas human studies typically do not—for example, they employ fixed location map reading tests, or at best, Virtual Reality (VR) devices to simulate the exploration of an environment while offering the experimental control of a laboratory setting. However, we already know that the act of moving is central to navigation. For example Ehinger et al. (2014) have shown that the integration of vestibular and kinesthetic information (provided through the navigation of the physical body in the environment) modulates brain activity in the alpha frequency band. These findings demonstrate that sensory and vestibular feedback are essential parts of spatial navigation that are neglected in lab-based navigation experiments. As a result, an obvious application of the mobile cognition approach is the study of the how we explore and navigate in real-world environments. We envisage participants navigating around complex environments (maze like corridors of large buildings, or around parks or city centers), performing route-finding tasks in the real world, whilst wearing a host of mobile cognition sensors. Such an approach would allow researchers to see whether there are human analogs of the phenomena seen in rats (e.g., place, head direction and speed signals), which to this point have only been assessed in virtual navigation tasks (Maguire et al., 1998; Ekstrom et al., 2003; Doeller et al., 2010; Jacobs et al., 2010; see Taube et al., 2013 for a critical review).

The mobile cognition approach is also particularly suited to investigating attention. Current understanding of attention is mainly based on visual exploration studies that have used static scenes (or at best moving images), while participants are stationary themselves. These studies necessarily place participants in a relatively passive spectating perspective, potentially over-emphasizing top-down influences on visual exploration. Indeed, the artificial nature of the stimuli or the task, and the restriction of participants' behavior, all inevitably lead to a very specific context that does not involve the same interaction between perception and action that can be found in complex and dynamic environments. The deployment of (visual) attention in the real-world may therefore be more sensitive to bottom-up influences, emphasizing the dynamic integration of information coming from multiple external and internal sources. Consider, for example, the role of attention when shopping: a large amount of information must be attended to in real-time—providing feedback that allows us to reorient our attention on elements of our surroundings that matter at that very moment, and allows us to adjust our movements to satisfy our goals. In this context, the interdependence between perception, cognition and action is clear, and forces attention to be considered alongside the integration of sensorimotor information, during interaction with our environment. Whether existing theoretical accounts of attention produced within laboratory settings can accommodate the varieties of attention found in real-world settings remains to be seen.

We believe that moving toward a mobile cognition framework will also lead to changes in the way that problems are approached. For example, in the context of motor cognition, we predict a move away from stereotyped, relatively narrow, response options, toward more complex, self-generated and spontaneous movement. On this basis, the investigation of sporting behavior can move away from examining the impact of sporting expertise on the performance of abstract laboratory based tests (e.g., demonstrating that the P300 elicited by auditory oddballs is larger in elite athletes). Instead athletes can be examined whilst performing real sports behavior (cf. Park et al., 2015), in real-world environments. Such an approach is more likely to deliver correlates of predictive value. Laboratory performance may not produce effective predictors of sporting performance, while mobile cognition should capture the highly adaptive and integrated complexity of sporting behavior, producing models with far greater applied relevance. Similarly, for health science, a mobile cognition approach could add considerable value by producing evidence-based interventions of real societal application. For example, we may be able to better predict (and therefore help prevent) falls in the elderly if models fully capture the multi-modal, integrative and environment-based nature of the problem—evident in the act of getting up from the chair to answer the door (for example, Nieuwboer et al., 2007). Equally, for rehabilitation following brain injury such as stroke, or when considering the consequences of dementia, it seems particularly important to have an understanding of the cognitive processes in the complex, dynamic, modality integrated reality of real-life settings.

The potential of a mobile cognition approach in terms of clinical applications is particularly likely to be far-reaching. One obvious first step is to examine problem behaviors, such as falls in the elderly, using real-world monitoring to capture the physiological and neural pre-cursors of relatively rare but critical behavioral errors. Monitoring brain states of patients at home may yield crucial information to devise and adjust informed medical decisions (e.g., stroke and epilepsy patients; Askamp and van Putten, 2014). The possibility to record brain activity during whole body motion and the related processing methods to handle motion artifacts allow to study populations that experience difficulties to remain still such as children and patients suffering from motor impairments (e.g., ALS, Parkinson's disease). In the future, mobile techniques may be integrated to cognitive rehabilitation strategies under the form of neurofeedback and the online acquisition of cognitive biomarkers metrics could be used by medical practitioners as tailored and ecological assessment tools to assist in the diagnosis and rehabilitation processes of patients affected by various neurological etiologies.

## Conclusion

Venturing away from highly controlled laboratories-based experiments opens up a range of new research questions, adding value to the traditional cognitive neuroscience approach. At the very least, the exploration of real-life cognition is likely to lead to the refinement or correction of previous models, while achieving greater ecological validity. At best, mobile cognition will remove barriers, allowing naturalistic behavior to be studied *in situ*, narrowing the explanatory gap between what is measured experimentally and what it means for understanding the human mind. To deliver on this promise inevitably implies tackling a number of methodological, technical and conceptual issues. Innovations in experimental design, processing and analyses methods will be required to address the major challenges of the study of human cognition in complex environments. More important, perhaps, moving to a mobile cognition approach requires an intellectual adjustment, away from an atomized examination of individual cognitive sub-processes, letting go of a degree of experimental control and ownership of isolated cognitive domains. Fundamentally, however, our view is that the greatest motivation for adopting a mobile cognition approach is simply the exciting prospect of developing a more relevant, ecologically valid, cognitive science.

## Author contributions

All authors listed, have made substantial, direct and intellectual contribution to the work, and approved it for publication.

### Conflict of interest statement

The authors declare that the research was conducted in the absence of any commercial or financial relationships that could be construed as a potential conflict of interest.
